# Constitutive Promoters Functional in Plant, Fungal, and Bacterial Hosts

**DOI:** 10.1021/acssynbio.4c00802

**Published:** 2025-05-16

**Authors:** Viktor V. Morozov, Anastasia V. Balakireva, Maxim M. Perfilov, Tatyana V. Chepurnykh, Ilia V. Yampolsky, Karen S. Sarkisyan, Alexander S. Mishin

**Affiliations:** † Planta LLC, Moscow 121205, Russia; ‡ Shemyakin-Ovchinnikov Institute of Bioorganic Chemistry, 68485 Russian Academy of Sciences, Moscow 119991, Russia; § Pirogov Russian National Research Medical University, Moscow 117997, Russia; ∥ Synthetic Biology Group, MRC London Institute of Medical Sciences, London W12 0HS, U.K.; ⊥ Institute of Clinical Sciences and Imperial Centre for Engineering Biology, 154297 Imperial College London, London UK W12 0NN, U.K.

**Keywords:** Synthetic promoter, synthetic terminator, constitutive promoter, bacteria, yeast, plant cells

## Abstract

Engineering of orthogonal systems functional across diverse hosts can benefit from employing universal regulatory DNA elements. Here, we screened a number of composite promoters in plant, fungal, and bacterial hosts, identifying variants that drive strong constitutive expression in *Nicotiana tabacum*, *Saccharomyces cerevisiae*, *Escherichia coli*, and *Agrobacterium tumefaciens*, or only in the eukaryotic subset of these organisms. These promoters can be used in universal vectors to co-optimize for different hosts in directed evolution, engineering of biosynthetic pathways, or other biotechnological tasks that require host switching.

## Graphical abstract

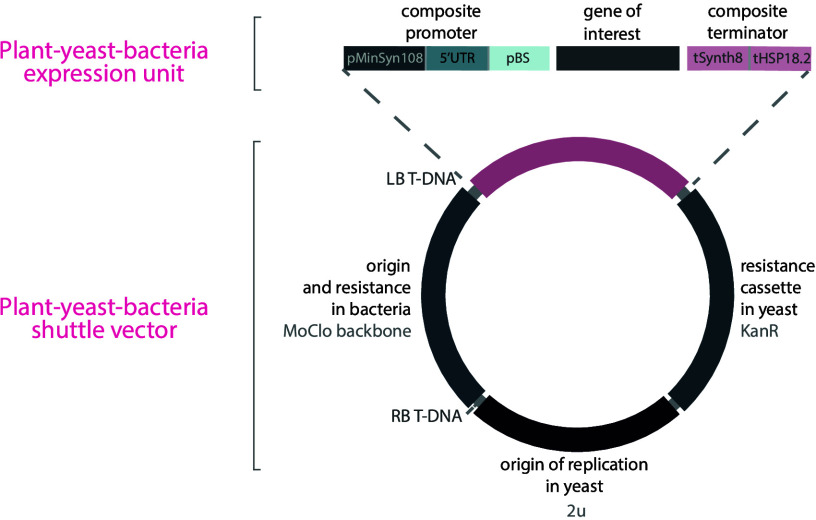

Just as in software, where the need to simplify and shorten development cycles drove generation of libraries with cross-platform functionality, reliance on DNA parts functional across different hosts can streamline bioengineering projects that require host switching.^1^ Previous efforts resulted in engineering of broad-spectrum promoters and shuttle vectors functional in *E. coli* and *P. pastoris*,^2^ *E. coli* and *S. cerevisiae*,^3^ animal and bacterial expression systems,^4^ and others. For our projects, we needed promoters active across plant, yeast and/or bacterial hosts, and we could not find such promoters in the literature.^5–7^ In this research, we focus on the development of promoters and terminators that function simultaneously in plant, yeast, and bacterial hosts. This advancement enables directed evolution experiments and screening assays across these platforms without the need for additional cloning.

We thus aimed to construct broad-spectrum promoters and to build vectors driving constitutive expression in these hosts. We chose a simple strategy of stacking various known promoters and 5′UTRs, hoping to identify a composite promoter variant with optimal expression in all our target hosts. Although these regulatory elements contained 5′UTRs, for simplicity, in this report we will refer to them as promoters. Briefly, we constructed eight promoter sequences with the following three architectures (Figure 1a): (1) pPlant — pYeast — 5′UTR_Plant; (2) pPlant — 5′UTR_Plant — pYeast; (3) pYeast — pPlant — 5′UTR_Plant. These promoters were paired with a composite terminator that we created by concatenation of yeast- and plant-active terminators tHSP18.2 or tSynth8 and tTDH1, respectively, and that we assumed to be active in both hosts.

**Figure 1:**
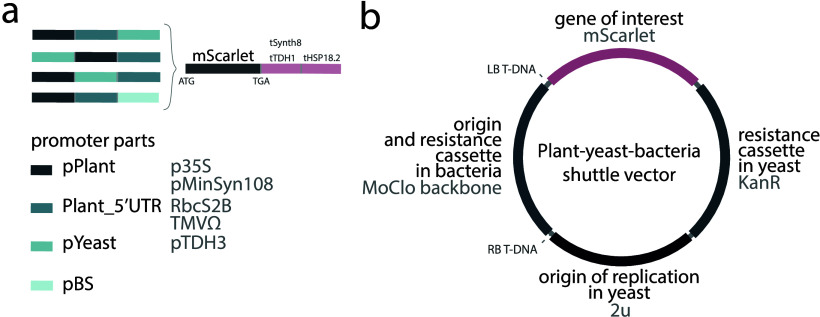
Composite promoters and vector structure. (a) Structure of mScarlet transcription unit containing composite promoters and terminators. (b) Structure of the vector used for the study and containing mScarlet gene controlled by the developed promoters and terminators, origin and resistance cassette in bacteria, resistance cassette in yeast, and origin of replication in yeast.

We chose a mix of natural and synthetic regulatory elements to construct promoters, aiming to test only a subset of possible stacking combinations for each architecture. Two plant promoters were tested: a 0.4-kbp variant of the cauliflower mosaic virus p35S promoter^8^ and a synthetic promoter, MinSyn108.^9^ These were combined with two plant 5′UTRs: tobacco mosaic virus omega (5′UTR_TMVΩ) or *Arabidopsis thaliana* Rubisco small subunit 2B gene (5′UTR_RbcS2B).^10^ Yeast promoters we tested included a natural *S. cerevisiae* glyceraldehyde-3-phosphate dehydrogenase promoter pTDH3, and a synthetic promoter pK528.^11^ Additionally, broad-spectrum promoter pBS^7^ active across yeast, Gram-negative and Gram-positive bacteria, was used as an alternative yeast-active promoter. Three transcription units were used as controls: plant-active p35S–5′UTR_RbcS2B–mScarlet–tHSP18.2, yeast-active pTDH3–mScarlet–tTDH1, and bacteria-active pTac–mScarlet–L3S2P21.

To test the variants, we assembled transcription units where each promoter controlled expression of mScarlet optimized for expression in *Nicotiana tabacum*, *Saccharomyces cerevisiae*, and *Escherichia coli*. We cloned these transcription units along with *Saccharomyces cerevisiae* 2-μm origin of replication and *S. cerevisiae* kanamycin resistance cassette into a binary MoClo Level 2 vector from.^12^ This backbone encoded a resistance cassette and origins of replication for both *E. coli* and *A. tumefaciens*. The vector also contained T-DNA borders that enabled the transfer of DNA into the nucleus of plant cells by agrobacteria (Figure 1b). We tested plasmids in four hosts: *Nicotiana tabacum* BY-2 cells, *Saccharomyces cerevisiae*, *Escherichia coli*, and *Agrobacterium tumefaciens*. For transient expression in BY-2 plant cells, we used an *Agrobacterium*-mediated transformation protocol. Some composite promoters and terminators may be functional in both *Agrobacterium* and plant cells. To differentiate the light emitted by BY-2 cell packs from that produced by the *Agrobacteria* used for transformation, we included a control in which plant cells were heat-inactivated before infection.

All composite promoters were active in plant cells (Figure 2). Promoters having the following architecture — pYeast–pPlant–5′UTR_Plant — generally showed the brightest signal. However, this same architecture did not perform well in yeast: only promoters where pTDH3 was placed *after* p35S or 5′UTR led to fluorescence. As expected, the pBS-containing promoter, which is active in *Agrobacterium* cells, enabled fluorescence in dead BY-2 cell samples. This indicates that the fluorescence originated from the *Agrobacterium* cells themselves and not the BY-2 cells.

**Figure 2:**
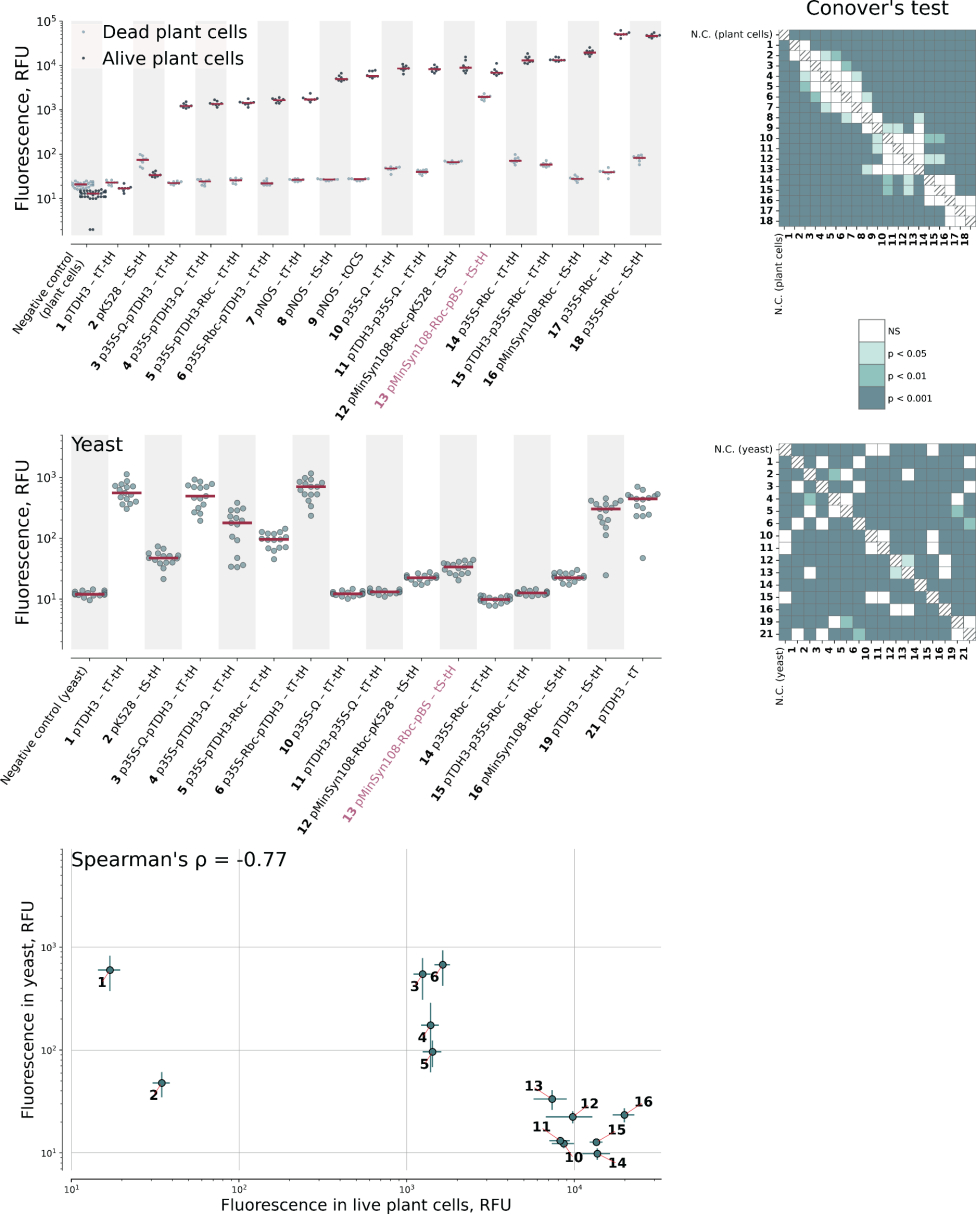
Performance of composite promoters in plant (*N. tabacum* BY-2 cells) and yeast (*S. cerevisiae*). mScarlet expression driven by developed promoters and terminators in plant cells (“alive plant cells, top), in *A. tumefaciens* applied to dead plant cells (“dead plant cells, top), in yeast cells (middle), and correlation of fluorescence signal in plants or yeast cells (bottom). The red lines are medians. Abbreviations: tS – tSynth8, tH – tHSP18.2, tT – tTDH1, Rbc – 5′UTR_RbcS2B, Ω – 5′UTR_TMVΩ. Plots are supplied with p-value of post-hoc two-sided Conover’s test corrected by the step-down method using Šidák adjustments (for plant cells only “alive plant cells” were statistically tested). Kruskal–Wallis H Test: H-statistic = 176.10, *p* = 3.9e-30 (plant cells), H-statistic = 2350.50, *p* < 1e-293. (yeast). On correlation plot data shown as a mean ± SD (in both dimensions).

Unexpectedly, we found that one of our controls, plant synthetic promoter pMinSyn108, drove strong expression not only in plant cells but also in yeast, on par with the synthetic yeast promoter (no. 16 in Figure 2). When coupled with the broad-spectrum pBS promoter, it enabled mScarlet expression across all four tested hosts (no. 13 in Figure 2). We also tested pMinSyn108 in *Pichia pastoris* and confirmed its activity. Analysis of pMinSyn108 sequence with http://www.yeastract.com/ service identified binding sites for 21 transcription factors from *S. cerevisiae*, potentially explaining its activity in yeast.^13^ Fifteen of these sites were also found in the sequence of the pTDH3 promoter.

To conclude, we report promoter variants active across plant and fungal hosts. When expression in yeast needs to be maximized, we recommend using promoter 6 (p35S–5′UTR_RbcS2B–pTDH3). If achieving the highest expression in plants is the priority, we recommend using bare pMinSyn108 with RbcS2B UTR (promoter 16, pMinSyn108–5′UTR_RbcS2B). Finally, if adding bacterial expression is desirable, promoter 13 can be used (pMinSyn108–5′UTR_RbcS2B–pBS), which is active in *E. coli*, *Agrobacterium tumefaciens*, *N. tabacum*, and *S. cerevisiae* cells that together with composite terminator tSynth8-tHSP18.2 makes them an optimal pair for gene expression in these systems. We expect these elements to find use in the construction of broad-spectrum selectable markers, engineering of universal expression vectors, synthetic genome projects, and other studies that require host switching.

## Materials and Methods

### Design and Assembly of Genetic Constructs

The coding sequence of mScarlet was optimized for expression in *E. coli*, *N.tabacum*, and *S.cerevisiae* and ordered synthetically, as well as promoters and terminators (Supplementary Table 1). Golden Gate assembly was performed in the T4 ligase buffer (Thermo Fisher) containing 10 U of T4 ligase, 20 U of either BsaI or BpiI (Thermo Fisher) and ∼100 ng of each DNA part. Typically, Golden Gate reactions were performed according to ‘troubleshooting’ cycling conditions described in^14^ 25 cycles (90 s at 37 °C, 180 s at 16 °C), then 5 min at 50 °C and 10 min at 80 °C.

Correct DNA assembly was typically confirmed by Sanger sequencing and in some cases additionally by Nanopore or Illumina-based whole plasmid sequencing. DNA assembly and whole-plasmid sequencing were typically ordered from the Cloning Facility (cloning.tech).

### Transformation of *Agrobacterium tumefaciens*

Plasmids were transformed into competent cells of *Agrobacterium tumefaciens* AGL0,^15^ and clones were selected on LB (Luria–Bertani) agar plates containing 50 mg/L of rifampicin and an additional antibiotic, depending on the plasmid used for transformation (200 mg L^–1^ of carbenicillin, 50 mg L^–1^ of kanamycin or 100 mg L^–1^ spectinomycin). Individual colonies were then inoculated into 10 mL of LB medium containing the same concentration of antibiotics. After overnight incubation at 28 °C with shaking at 220 rpm, cultures were centrifuged at 2,900g, resuspended in 25% glycerol, and stored as glycerol stocks at −80 °C.

### Validation of Reporters in Plant Cell Packs Based on *Nicotiana tabacum* BY-2 Cell Culture

BY-2 cell culture was grown in BY-2 medium (Murashige and Skoog (MS) with 0.2 mg L^–1^ 2,4-dichlorophenoxyacetic acid, 200 mg L^–1^ KH_2_PO_4_, 1 mg L^–1^ thiamine, 100 mg L^–1^ myo-inositol, and 30 g L^–1^ sucrose) at 27 °C by shaking at 130 rpm in darkness, with 2 mL of 1-week-old culture being transferred into new 200 mL of BY-2 medium every week.^16^

Transformations of BY-2 cell packs were made according to a protocol adapted from.^17^ One-week-old BY-2 culture was pelleted in black 96-well plates to create cell packs that were infiltrated by a mixture of several agrobacterial strains containing binary vectors. One of the strains encoded silencing inhibitor P19 (OD_600_0.2), and the other encoded mScarlet transcription unit (OD_600_0.5).

Inactivation of BY-2 cell packs was performed by heating the plates for 10 min at 85 °C.

### Transformation of *Saccharomyces cerevisiae*

The protocol for cultivation and transformation of yeast *Saccharomyces cerevisiae* was obtained from ref 18. The plasmids were meant for episomal expression and carried a resistance cassette to kanamycin. Plasmids were used for the transformation of electrically competent yeast cells. Colonies were selected using 200 mg mL^–1^ of G418. Polymerase chain reaction (PCR)-based screening of yeast colonies was done by heating up colonies in 10 μL of 20 mM NaOH at 90 °C for 7 min and then using 1 μL of the resulting solution for direct PCR.

### Fluorescence Imaging

Plates with BY-2 cells were incubated at 80% humidity at 22 °C and imaged after 48 h since the start of expression using the TECAN Spark imager, at excitation wavelength 555 nm and emission wavelength 601 nm. Agrobacteria were imaged the same way. *E. coli* and yeast colonies were imaged using IVIS Spectrum In vivo imager at 1 s exposure at excitation wavelength 575 and emission wavelength 600.

Data presentation and statistical analysis Most of the data are plotted as medians and colored individual data points using Seaborn (https://seaborn.pydata.org/, ver. 0.13.2) and Matplotlib (https://matplotlib.org/, ver. 3.8.0) packages, using Python version 3.10.12. Kruskal–Wallis H tests (scipy.stats package, https://www.scipy.org/, SciPy version 1.13.1) followed (H_0_ was rejected) by multiple pairwise posthoc two-sided Conover’s tests (Scikit-posthocs package,^19^ version 0.10.0) with P values corrected by the step-down method using Šidák adjustments were computed. The Spearman correlation coefficient (Figure 1) was calculated using Pandas functions (https://pandas.pydata.org/, ver. 2.2.2). Sample numbers (N) are reported in the figures.

## Supplementary Material



## Data Availability

The plasmids used in this study will be made available upon request.
