# Supplementary material for: Constitutive Promoters Functional in Plant, Fungal, and Bacterial Hosts
Source: ACS Synth Biol. 2025 May 16;14(6):2375–9. doi: 10.1021/acssynbio.4c00802 (PMC12186668; doi:10.1021/acssynbio.4c00802)
Supplement: Supplementary file 1 [file sb4c00802_si_001.pdf]

Supporting information

Supplementary Table 1. Plasmids used in the study.

Constitutive promoters functional in plant, fungal and bacterial hosts

Viktor V Morozov[*2], Anastasia V Balakireva[*1,2], Maxim M Perfilov[1,2], Tatyana V. Chepurnykh[2], Ilia V Yampolsky[1,2,3], Karen S Sarkisyan[#4,5] and Alexander S Mishin[#1,2]
[*] equal contribution, [#] corresponding authors (alexander@planta.bio, karen.s.sarkisyan@gmail.com)

[1] Planta LLC, Moscow, Russia, 121205
[2] Shemyakin-Ovchinnikov Institute of Bioorganic Chemistry, Russian Academy of Sciences, Moscow, Russia, 117997
[3] Pirogov Russian National Research Medical University, Moscow, Russia, 117513
[4] Synthetic Biology Group, MRC London Institute of Medical Sciences, London, UK, W12 0HS
[5] Institute of Clinical Sciences, Faculty of Medicine, Imperial College London, London, UK, SW7 2AZ

#	Assayed transcription unit	Plasmid structure	Link to the map
1	pTDH3 – mScarlet – tTDH1–tHSP18.2	L2 | pTDH3 – mScarlet – tTDH1–tHSP18.2 | ori_sc_2_micron_origin | pTEF1 - scKanR - tTEF1	https://benchling.com/s/seq-syi7v3RLv8FyFPRS9pfa?m=slm-dX9n6NVfaKG7kXGmxHbS
2	pK528 – mScarlet – tSynth8–tHSP18.2	L2 | pK528 – mScarlet – tSynth8–tHSP18.2 | ori_sc_2_micron_origin | pTEF1 - scKanR - tTEF1	https://benchling.com/s/seq-4PqzcTaWvCseR7j2b2Th?m=slm-Z7MxEfaJ8H2SKGlyM7h7
4	p35S–5'UTR_TMVΩ–pTDH3 – mScarlet – tTDH1–tHSP18.2	L2 | p35S–5'UTR_TMVΩ–pTDH3 – mScarlet – tTDH1–tHSP18.2 | ori_sc_2_micron_origin | pTEF1 - scKanR - tTEF1	https://benchling.com/s/seq-Skg0AqEhBlOqarpMyXb7?m=slm-gpggwUuoP1G6U7Ymidwg
5	p35S–pTDH3–5'UTR_RbcS2B – mScarlet – tTDH1–tHSP18.2	L2 | p35S–pTDH3–5'UTR_RbcS2B – mScarlet – tTDH1–tHSP18.2 | ori_sc_2_micron_origin | pTEF1 - scKanR - tTEF1	https://benchling.com/s/seq-Qa2ntdOc24VA4X7A0K0o?m=slm-BbVZIEcjpMQZIklngv6Y
6	p35S–5'UTR_RbcS2B–pTDH3 – mScarlet – tTDH1–tHSP18.2	L2 | p35S–5'UTR_RbcS2B–pTDH3 – mScarlet – tTDH1–tHSP18.2 | ori_sc_2_micron_origin | pTEF1 - scKanR - tTEF1	https://benchling.com/s/seq-35cq4SJf3rCfpoz6SDDz?m=slm-M7qsP85BQbpqZMqW1ARw
7	pMinSyn108–5'UTR_RbcS2B–pBS–RBS – mScarlet – tSynth8–tHSP18.2	L2 | pMinSyn108–5'UTR_RbcS2B–pBS–RBS – mScarlet – tSynth8–tHSP18.2 | ori_sc_2_micron_origin | pTEF1 - scKanR - tTEF1	https://benchling.com/s/seq-H8RhLnGScyky5YsTst2g?m=slm-iypCZfWhtrzq8f1p9iJ8

8	pMinSyn108–5'UTR_RbcS2B–pK528 – mScarlet – tSynth8–tHSP18.2	L2 | pMinSyn108–5'UTR_RbcS2B–pK528 – mScarlet – tSynth8–tHSP18.2 | <br>ori_sc_2_micron_origin | pTEF1 - scKanR - tTEF1	https://benchling.com/s/seq-PFinkceEwzOPL0Y8QPrL?m=slm-Phl8ZvnQPdtVyTeo2lu1
9	p35S–5'UTR_TMVΩ – mScarlet – tTDH1–tHSP18.2	L2 | p35S–5'UTR_TMVΩ – mScarlet – tTDH1–tHSP18.2 | <br>ori_sc_2_micron_origin | pTEF1 - scKanR - tTEF1	https://benchling.com/s/seq-mDELec8mL9FrsIKLAKuV?m=slm-l1DpRQHdHljWSBWNpCxK
10	pTDH3–p35S–5'UTR_TMVΩ – mScarlet – tTDH1–tHSP18.2	L2 | pTDH3–p35S–5'UTR_TMVΩ – mScarlet – tTDH1–tHSP18.2 | <br>ori_sc_2_micron_origin | pTEF1 - scKanR - tTEF1	https://benchling.com/s/seq-n3vtoIUBHABNtp4QP2eV?m=slm-XiGRnYo6ksKRr9zfTQ2c
11	p35S–5'UTR_RbcS2B – mScarlet – tTDH1–tHSP18.2	L2 | p35S–5'UTR_RbcS2B – mScarlet – tTDH1–tHSP18.2 | <br>ori_sc_2_micron_origin | pTEF1 - scKanR - tTEF1	https://benchling.com/s/seq-e9nHD68K6SKnA0fHVQ4k?m=slm-Xh3rYn2DuhEIUbJ5TkEp
12	pMinSyn108–5'UTR_RbcS2B – mScarlet – tSynth8–tHSP18.2	L2 | pMinSyn108–5'UTR_RbcS2B – mScarlet – tSynth8–tHSP18.2 | <br>ori_sc_2_micron_origin | pTEF1 - scKanR - tTEF1	https://benchling.com/s/seq-TjgjxrkynoYg9Cia5sC6?m=slm-NB9vcbVca8rM53TXxhYQ
13	pTDH3–p35S–5'UTR_RbcS2B – mScarlet – tTDH1–tHSP18.2	L2 | pTDH3–p35S–5'UTR_RbcS2B – mScarlet – tTDH1–tHSP18.2 | <br>ori_sc_2_micron_origin | pTEF1 - scKanR - tTEF1	https://benchling.com/s/seq-DwgYkWDbg0dN51PnBK65?m=slm-pnxOXL6EGrJJ9rhKExNw
14	pTac - mScarlet - tL3S2P21 | ori_sc_2_micron_origin | p_TEF1 - scKanR - t_TEF1	L2 | pTac - mScarlet- tL3S2P21 | ori_sc_2_micron_origin | p_TEF1 - scKanR - t_TEF1	https://benchling.com/s/seq-F2UIMeXdskAARq9ZScAD?m=slm-TdIuA1jtNxn4d05Xbm8I
15	pTac - mScarlet - tTDH1 - tHSP18.2 | ori_sc_2_micron_origin | p_TEF1 - scKanR - t_TEF1	L2 | pTac - mScarlet - tTDH1 - tHSP18.2 | ori_sc_2_micron_origin | p_TEF1 - scKanR - t_TEF1	https://benchling.com/s/seq-Qqyft6LSeypuw3LfSTNR?m=slm-ZjahfjHE2uJ6tN3SgZ8T
16	pTac - mScarlet - tSynth8 - tHSP18.2 | ori_sc_2_micron_origin | p_TEF1 - scKanR - t_TEF1	L2 | pTac - mScarlet - tSynth8 - tHSP18.2 | ori_sc_2_micron_origin | p_TEF1 - scKanR - t_TEF1	https://benchling.com/s/seq-0KeIysZmigBDgeNzX7jk?m=slm-8px4TLqDWf0ypCDss5UU

17	p35S - 5'UTR_RbcS2B - mScarlet - tHSP18.2 | ori_sc_2_micron_origin | p_TEF1 - scKanR - t_TEF1	L2 | p35S - 5'UTR_RbcS2B - mScarlet - tHSP18.2 | ori_sc_2_micron_origin | p_TEF1 - scKanR - t_TEF1	https://benchling.com/s/seq-fkTMJWwRYBn3XK9j80Ia?m=slm-vbke62HTO0Ku10B78NEp
18	pTDH3 - mScarlet - tTDH1 | ori_sc_2_micron_origin | p_TEF1 - scKanR - t_TEF1	L2 | pTDH3 - mScarlet - tTDH1 | ori_sc_2_micron_origin | p_TEF1 - scKanR - t_TEF1	https://benchling.com/s/seq-lISzEcqdtzzD2XaMb8po?m=slm-pip3zdEXOjUjhdsivjyy
19	pTDH3 - mScarlet - tSynth8 - tHSP18.2 | ori_sc_2_micron_origin | p_TEF1 - scKanR - t_TEF1	L2 | pTDH3 - mScarlet - tSynth8 - tHSP18.2 | ori_sc_2_micron_origin | p_TEF1 - scKanR - t_TEF1	https://benchling.com/s/seq-2ygYdqg4kniBACCpvrEA?m=slm-2kPE03lm4KHl7MnMBqow
20	p35S - 5'UTR_RbcS2B - mScarle - tSynth8 - tHSP18.2 | ori_sc_2_micron_origin | p_TEF1 - scKanR - t_TEF1	L2 | p35S - 5'UTR_RbcS2B - mScarle - tSynth8 - tHSP18.2 | ori_sc_2_micron_origin | p_TEF1 - scKanR - t_TEF1	https://benchling.com/s/seq-RVDbVYxEE8Rha0ThSWCK?m=slm-rSUix02nEh58WYRhXQkx

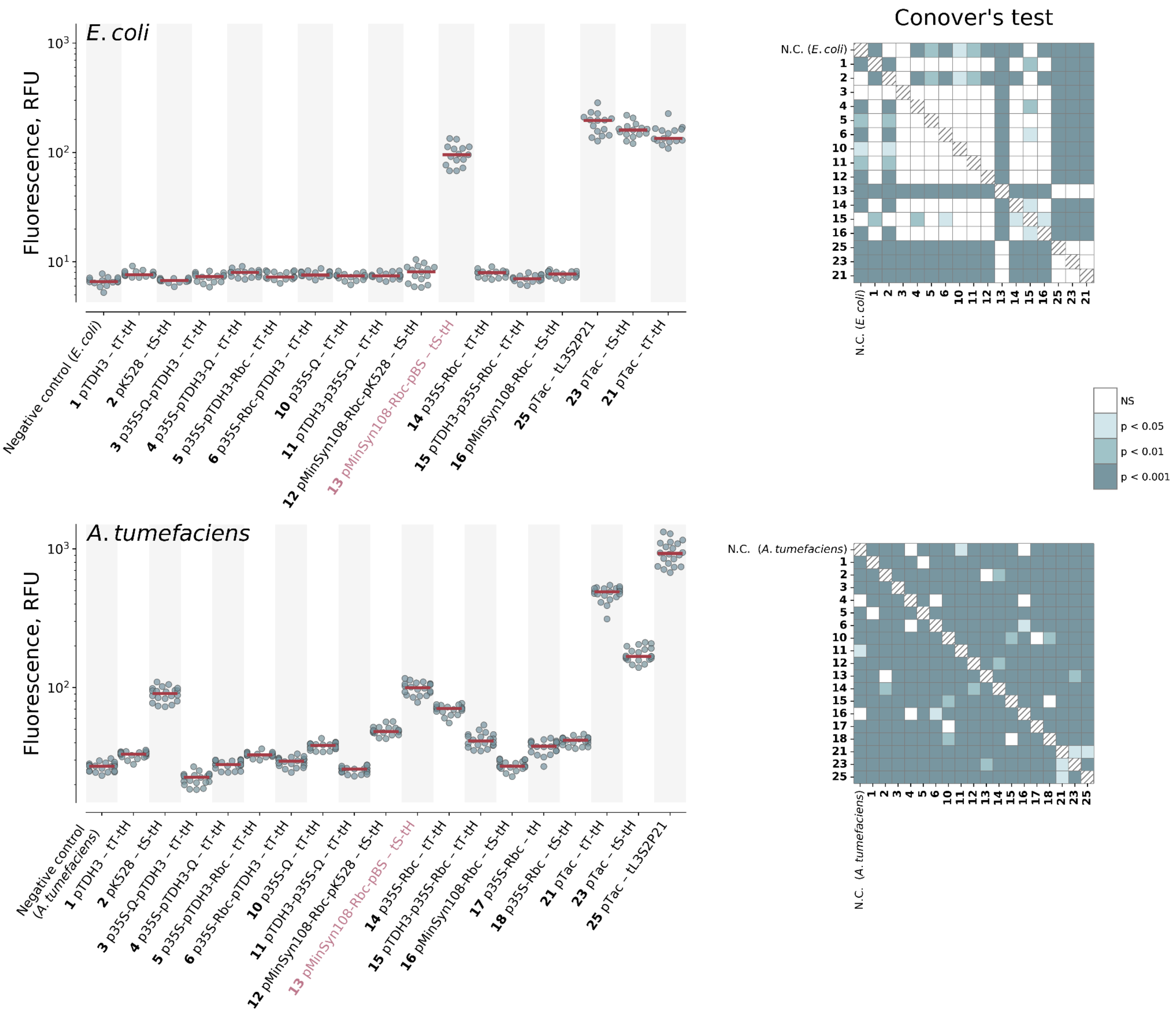

Constitutive promoters functional in plant, fungal and bacterial hosts

Viktor V Morozov* [2], Anastasia V Balakireva* [1,2], Maxim M Perfilov [1,2], Tatyana V. Chepurnykh [2], Ilia V Yampolsky [1,2,3], Karen S Sarkisyan# [4,5] and Alexander S Mishin# [1,2]

* equal contribution, # corresponding authors (alexander@planta.bio, karen.s.sarkisyan@gmail.com)

[1] Planta LLC, Moscow, Russia, 121205
[2] Shemyakin-Ovchinnikov Institute of Bioorganic Chemistry, Russian Academy of Sciences, Moscow, Russia, 117997
[3] Pirogov Russian National Research Medical University, Moscow, Russia, 117513
[4] Synthetic Biology Group, MRC London Institute of Medical Sciences, London, UK, W12 0HS
[5] Institute of Clinical Sciences, Faculty of Medicine, Imperial College London, London, UK, SW7 2AZ

Supplementary figure 1. Performance of composite promoters in *E.coli* and *A.tumefaciens*. The red lines are medians. Abbreviations: tS – tSynth8, tH – tHSP18.2, tT – tTDH1, Rbc – 5'UTR_RbcS2B, Ω – 5'UTR_TMVΩ. Plots are supplied with p-value of post-hoc two-sided Conover's test corrected by the step-down method using Šidák adjustments (for plant cells only "alive plant cells" were statistically tested). Kruskal-Wallis H Test: H-statistic = 11311.14, $p < 1e-293$ (*E.coli*), H-statistic = 75.60, $p = 1.8e-10$ (*A.tumefaciens*).
